# Subwavelength imaging through ion-beam-induced upconversion

**DOI:** 10.1038/ncomms9832

**Published:** 2015-11-12

**Authors:** Zhaohong Mi, Yuhai Zhang, Sudheer Kumar Vanga, Ce-Belle Chen, Hong Qi Tan, Frank Watt, Xiaogang Liu, Andrew A. Bettiol

**Affiliations:** 1 Department of Physics, Centre for Ion Beam Applications, National University of Singapore, Singapore 117542, Singapore; 2 Department of Chemistry, National University of Singapore, Singapore 117543, Singapore; 3 Institute of Materials Research and Engineering, Agency for Science, Technology and Research, Singapore 117602, Singapore; 4 Center for Functional Materials, NUS (Suzhou) Research Institute, Suzhou, Jiangsu 215123, China; 5 Yale-NUS College, Singapore 138527, Singapore

## Abstract

The combination of an optical microscope and a luminescent probe plays a pivotal role in biological imaging because it allows for probing subcellular structures. However, the optical resolutions are largely constrained by Abbe's diffraction limit, and the common dye probes often suffer from photobleaching. Here we present a new method for subwavelength imaging by combining lanthanide-doped upconversion nanocrystals with the ionoluminescence imaging technique. We experimentally observed that the ion beam can be used as a new form of excitation source to induce photon upconversion in lanthanide-doped nanocrystals. This approach enables luminescence imaging and simultaneous mapping of cellular structures with a spatial resolution of sub-30 nm.

## Abstract (web summary)

Combining high-resolution microscopic techniques with luminescent probes is important for biological imaging. Here, Mi *et al*. demonstrate subwavelength imaging by combining lanthanide-doped upconversion nanocrystals with ionoluminescence, revealing cellular structure and particle spatial distribution at high resolution.

## Graphical abstract

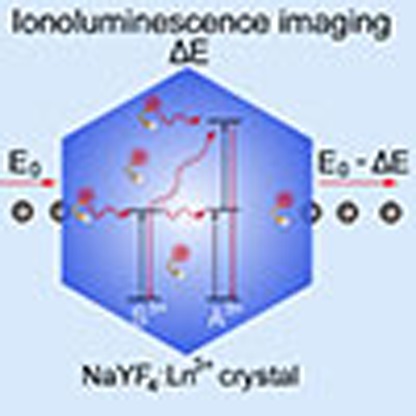

Photoluminescent probes play an indispensable role in labelling and manipulating biological species for many areas of application such as molecular imaging at the subcellular level^1^,^2^,^3^,^4^, *in vivo* biodetection^5^,^6^,^7^,^8^ and targeted intracellular delivery of therapeutics^9^,^10^. In particular, lanthanide-doped upconversion nanocrystals^11^,^12^,^13^ have recently gained considerable attention for use as biomarkers owing to their unique ability to convert low-energy light into high-energy photons, coupled with the absence of photobleaching and photoblinking^14^,^15^. However, an infrared laser, either in continuous- or pulsed-wave mode, is generally needed to implement photon upconversion^16^,^17^,^18^,^19^,^20^. The use of the laser as the excitation source inevitably imposes an inherent constraint for high-resolution imaging because of Abbe's diffraction limit.

It has been well established that hexagonal-phase NaYF_4_ is one of the most efficient host materials frequently utilized for preparing upconversion nanocrystals^12^,^21^. The upconversion nanocrystals are typically doped with ytterbium (Yb^3+^) sensitizer ions, which absorb infrared radiation centring at 980 nm and non-radiatively transfer their absorption to activator ions such as thulium (Tm^3+^), erbium (Er^3+^) or holmium (Ho^3+^). The notable prospects of lanthanide-doped nanocrystals, including non-photobleaching, tunable emission wavelength and controllable particle size^12^,^14^, have provided new opportunities for bioimaging applications in a variety of research fields^6^,^7^,^22^. In addition, the excitation of these nanocrystals in the near-infrared region eliminates background autofluorescence^6^,^8^. However, these imaging studies have been unable to provide detailed information on the single-particle level owing to the diffraction limit of infrared excitation light associated with conventional or even confocal microscope setups^23^,^24^.

To overcome the diffraction limit, a variety of optical super-resolution techniques, for instance stimulated-emission depletion microscopy^25^, have been developed. Alternatives to these super-resolution techniques are methods involving charged particles such as electron or ion beams, with the benefit of rendering much shorter de Broglie wavelengths. For example, electron microscope-based cathodoluminescence has been successfully utilized for high-resolution luminescence imaging^26^,^27^. However, the electrons suffer from large angle scattering when interacting with biological samples, which in turn compromises the resolution, especially for tissue imaging at a substantial depth.

Here we report, for the first time, the observation of photon upconversion through excitation of lanthanide-doped nanocrystals under a beam of helium ions. The use of mega-electron-volt- (MeV) focused helium ions offer significant advantages, as they can penetrate much thicker biological samples (up to several microns) with very little deviation in their trajectories^28^ (see Supplementary Fig. 1). We thus reason that the MeV-focused ion beam may serve as a new form of excitation source to induce photon upconversion in lanthanide-doped nanocrystals and, more importantly, to allow for high-resolution luminescence imaging beyond the diffraction limit.

## Results

### Construction of ion-beam imaging setup

**Figure 1:**
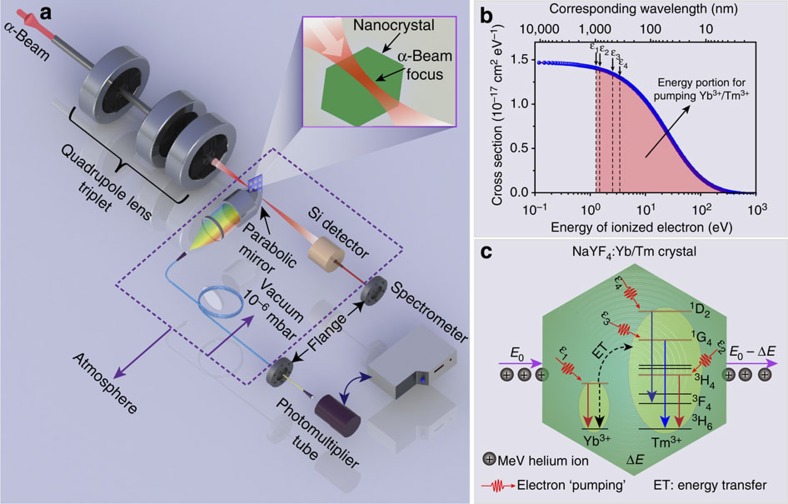
Experimental setup and proposed ionoluminescence mechanism. (**a**) Artist's view of the basic experimental setup. The focused beam with a spot size of sub-30 nm features can be achieved using a spaced triplet of compact magnetic quadrupole lenses. A Si surface barrier detector is equipped for measuring the energy loss distribution of the ions. (**b**) Calculated energy distribution of the ionized electrons by bombarding the MeV α-particles on the lanthanide-doped nanocrystals, showing different cross-sections of the resulting electrons at specific energies. Note that most of the ionized electrons have energies mainly located in the visible and infrared spectral region. (**c**) Proposed upconversion mechanism under α-beam irradiation. The incident helium ions with energy of *E*_0_ deposit a certain amount of energy (Δ*E*) onto the crystal to cause the atomic ionization inside the crystal. Subsequently, the ionized secondary electrons can release their energy, most likely during the electron-hole recombination process and successively transfer the energy to Yb^3+^ and Tm^3+^. An energy transfer from the excited Yb^3+^ to its neighbouring Tm^3+^ ions then populates the excited states (for example, ^3^H_4_, ^1^G_4_ and ^1^D_2_) of Tm^3+^.

The basic experimental setup is shown in Fig. 1a. A beam of 1.6 MeV helium ions (α-particles) is produced by a Singletron ion accelerator. A sample comprising NaYF_4_:Yb/Tm nanorods is placed in a vacuum chamber (10^−6^ mbar) at a position situated exactly along the beam path. A customized double-piece parabolic mirror with front and rear openings is used to collect emission photons induced by the ion beam and, concurrently, allow the ion beam to pass through the mirror (Supplementary Fig. 1). The convergent lens-coupled parabolic mirror allows the emitted light to be focused into a fibre, which guides the light out of the vacuum chamber. The emitted photons are then captured either by a photomultiplier tube for luminescence imaging or by a spectrometer for spectroscopic characterization. A Si surface barrier detector is used to perform scanning transmission ion microscopy imaging by measuring the energy loss during the penetration of the ions into a given sample^28^,^29^.

The inelastic collision of helium ions with atomic electrons in a crystal can lead to energy loss dominated through an excitation and atomic ionization process^30^. To understand the efficacy of the ionization in the NaYF_4_:Yb/Tm nanocrystal for photon upconversion, we first performed simulations on the energy distribution of the ionized secondary electrons using a Hansen–Kocbach–Stolterfoht theoretical model (Supplementary Notes)^30^. Our simulation result shows that the ionized electrons with energies larger than 1.265 eV (equivalent to 980 nm) hold a large portion (estimated to be 97.5%) of the total cross-sections (Fig. 1b). Thus, the ionized electrons within this energy portion can potentially be utilized by the Yb/Tm co-doped nanocrystal. On the basis of the energy-matching principle, we propose an energy transfer mechanism that governs the photon upconversion in the NaYF_4_:Yb/Tm nanocrystal system (Fig. 1c and Supplementary Fig. 2). It should be pointed out that the ionized electrons with energies higher than 1.265 eV may partially lose their energy by ionization, collision or phonon-coupling processes to match the energy levels of Yb^3+^ or Tm^3+^ for effective upconversion pumping.

### Spectroscopic study of lanthanide-doped crystals

**Figure 2:**
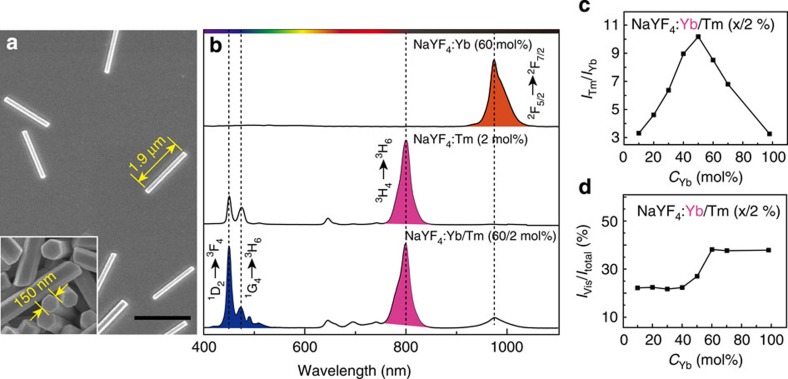
Spectroscopic analysis of ionoluminescence. (**a**) Scanning electron microscopy (SEM) image of the as-synthesized Yb^3+^/Tm^3+^-co-doped NaYF_4_ nanorods under investigation. The inserted high-resolution SEM image shows hexagonal cross-sections of these nanorods. Scale bar, 2 μm. (**b**) Comparative emission spectra of the NaYF_4_-based nanorods with different dopant compositions when irradiated with α-particles. (**c**) The plot of the emission ratio of Tm^3+^ and Yb^3+^ (*I*_Tm_/*I*_Yb_) as a function of Yb^3+^ doping concentration, supporting the energy transfer between the two lanthanide ions. (**d**) Optimization of Yb^3+^ doping concentration for maximal emission output in the visible range. The ratio of *I*_Vis_/*I*_total_ represents the percentage of integrated visible emission in the total emission covering the range from 350 to 1,100 nm.

To validate our hypothesis, we prepared a set of NaYF_4_-based nanorods with different dopant compositions through a hydrothermal procedure^23^ and systematically investigated their response to α-particle irradiation. Scanning electron microscopic imaging revealed the formation of monodisperse nanorods with an average size of 1.9 μm × 150 nm (Fig. 2a and Supplementary Figs 3 and 4). When singly-doped with Yb^3+^ (60 mol%) as the activator, the nanorods gave rise to emission at 975 nm on α-particle excitation, corresponding to ^2^F_5/2_→^2^F_7/2_ transition of Yb^3+^ (Fig. 2b). In contrast, NaYF_4_:Tm (2 mol%) nanorods exhibited an intense emission at 800 nm and two weak emissions at 450 and 480 nm, corresponding to ^3^H_4_→^3^H_6,_^1^D_2_→^3^F_4_ and ^1^G_4_→^3^H_6_ optical transitions of Tm^3+^, respectively. These results clearly verify that both Yb^3+^ and Tm^3+^ ions can directly harvest the energy of the ionized electrons upon the excitation with the α-particles. Intriguingly, in the case of NaYF_4_ nanorods co-doped with Yb/Tm (60/2 mol%), the blue emissions at 450 and 480 nm of Tm^3+^ showed a considerable enhancement, suggesting that the addition of Yb^3+^ in the NaYF_4_:Tm nanorods promotes ion-beam-induced upconversion emission of Tm^3+^ at short wavelengths through energy transfer upconversion.

To shed more light on the energy transfer between Yb^3+^ and Tm^3+^, we prepared a series of NaYF_4_:Yb/Tm nanorods with varied Yb^3+^ doping concentrations (10–98 mol%). We collected their luminescence spectra (Supplementary Figs 5 and 6) under α-particle irradiation and integrated the overall emission intensity for Tm^3+^ and Yb^3+^ ions, respectively. The intensity ratios of *I*_Tm_/*I*_Yb_, plotted against Yb doping content, was used to show the relative intensity change in Tm^3+^ and Yb^3+^ emissions. As shown in Fig. 2c, the measured intensity ratio of *I*_Tm_/*I*_Yb_ increased from 3.3 to 10.2 with the increase in Yb^3+^ concentration from 10 to 50 mol% and then decreased to 3.3 at a Yb^3+^ concentration of 98 mol%. Such inverse-parabolic profile provides a strong evidence for the energy transfer between Yb^3+^ and Tm^3+^. Particularly, the rising stage of *I*_Tm_/*I*_Yb_ indicates the occurrence of efficient energy transfer from Yb^3+^ to Tm^3+^, thus resulting in the pronounced enhancement of upconversion emission in short wavelengths. The descending stage of *I*_Tm_/*I*_Yb_ can be ascribed to the back-energy-transfer from Tm^3+^ to Yb^3+^ at high Yb^3+^ concentrations, analogous to the scenario in photon upconversion process in which a 980 nm laser (Supplementary Fig. 7 and Supplementary Table 1) is employed as the excitation source^23^.

### Luminescence imaging

**Figure 3:**
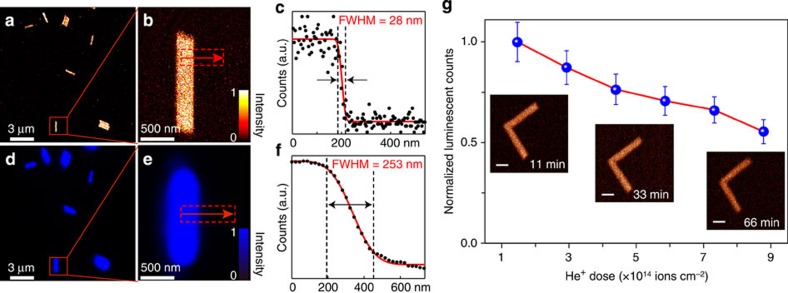
Luminescence imaging of NaYF_4_:Yb/Tm (60/2 mol%) nanorods. (**a**) Ionoluminescence image of the as-synthesized nanorods through α-particle excitation. (**b**) High-magnification ionoluminescence image of a single nanorod as marked in **a**. (**c**) The corresponding line-scanning profile extracted from the intensity counting at the region marked in **b** along the arrow, indicating an imaging resolution of about 28 nm. (**d**) Photoluminescence image of the same sample taken by using 980 nm laser excitation. (**e**) High-magnification photoluminescence image of the same nanorod as shown in **b**. (**f**) The corresponding line-scanning profile from the image shown in **e** showing a diffraction-limited resolution of 253 nm associated with conventional upconversion microscopes. (**g**) Ionoluminescence intensity profile as a function of the accumulated dosage of helium ions showing the considerable iono-bleaching resistance of the nanorods. The inserted images, taken at different time intervals (11, 33 and 66 min), indicate that the emission brightness of the nanorods remains essentially unaltered over time. Scale bars, 500 nm. The error bar represents the standard deviation of luminescence counts obtained from a single nanorod in two separate measurements.

High-resolution imaging can be achieved through α-beam irradiation of lanthanide-doped nanomaterials because the spot size of α-beam can be readily focused down to sub-30 nm (refs 28, 29). Considering that the spectral-response range of the photodetector used falls within the visible spectrum, we have adopted Yb^3+^/Tm^3+^ (60/2 mol%) as the optimal combination for maximal visible emission (Fig. 2d and Supplementary Fig. 6). Images of the NaYF_4_:Yb/Tm (60/2 mol%) nanorods were recorded in a 512 × 512 pixel array at a count rate of around 15,000 helium ions per second by detecting the α-particle-induced luminescence (Fig. 3a,b). To ascertain the spatial resolution of the ionoluminescence image, a representative line-scanning profile of an individual nanorod was collected and presented in Fig. 3c. By fitting the profile using a modified Gaussian model^31^, the imaging resolution of the α-particle-based ionoluminescence technique was determined to be 28 nm as defined by full-width at half maximum. By comparison, conventional optical microscopies equipped with a 980-nm diode laser showed a resolution limit of ∼253 nm (Fig. 3d–f and Supplementary Fig. 8). It should be noted that the effect of iono-bleaching, typically associated with the reduction in emission intensity in dye- or quantum dot-based systems^32^,^33^, does not pose a constraint to lanthanide-doped nanomaterials (Fig. 3g and Supplementary Figs 9 and 10).

## Discussion

**Figure 4:**
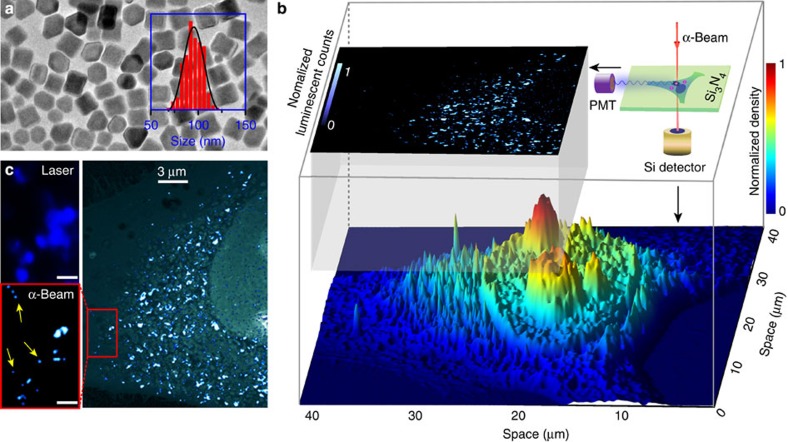
Particle-distribution mapping by ionoluminescence imaging. (**a**) Transmission electron microscopy image of the as-synthesized NaYF_4_:Yb/Tm (60/2 mol%) nanoparticles. The inserted histogram shows the size distribution of these nanoparticles. (**b**) Basic experimental design for structural determination and ionoluminescence imaging of the HeLa cell after uptake of the nanoparticles. The luminescence mapping of the nanoparticles and the 3D rendering of detailed cellular structures can be simultaneously implemented by capturing α-particle-induced photons through a photomultiplier tube (PMT) and by scanning transmission ion microscopy, respectively. (**c**) Comparative photoluminescence (top left) and ionoluminescence (right) imaging, with the latter clearly showing the ability to resolve single nanoparticles (marked by the arrows as shown in the magnified image). Note that top-left and bottom-left images are taken from the same section of the cell. Scale bars, 1 μm. Note that the photoluminescence image was generated by using a 980-nm-diode laser (Supplementary Fig. 12).

Importantly, the combination of scanning transmission ion microscopy and the α-particle-induced luminescence technique enables simultaneous structural determination and luminescence imaging on a single-cell level. As a proof of concept, we prepared NaYF_4_:Yb/Tm (60/2%) nanoparticles (∼95 nm) and incubated them with Human cervical carcinoma cells, which were seeded on a 100-nm-thick silicon-nitride membrane (Fig. 4a,b and Supplementary Fig. 11). By detecting the energy loss of transmitted ions through a Si surface barrier detector, we were able to generate an areal density map of a whole HeLa cell by scanning transmission ion microscopy, which provides detailed information on cellular structures (Fig. 4b). Concurrently, α-particle-induced photons were captured by a photomultiplier tube for luminescence mapping of the nanoparticles (Fig. 4b). The coupling of α-particle-induced luminescence imaging with scanning transmission ion microscopy allowed us to precisely locate the nanoparticles within the whole cell (Fig. 4c). Remarkably, single nanoparticles after cellular internalization could be resolved by our technique (see the enlarged panel in Fig. 4c). This was in stark contrast with the limit of resolution achievable by a conventional microscope equipped with a 980-nm diode laser (Fig. 4c, top-left panel). In the latter case, the photoluminescence imaging from the same area showed much reduced resolution (Supplementary Fig. 12).

Our findings could influence the study of the dynamics of upconversion processes and provide a better understanding of energy transfer in lanthanide-doped materials systems where the source of excitation may play a crucial role. The results presented here suggest that a sub-30 nm imaging resolution for upconversion nanocrystals is achievable through the use of α-particle-induced secondary electrons. By combining upconversion luminescence with scanning transmission ion microscopy, we have been able to map the distribution of individual nanoparticles within a whole cell and simultaneously reveal the 3D cellular structure at ultrahigh spatial resolution. This methodology will enable important applications in probing biological and biomedical processes at the subcellular level, for example, the quantitative measurement of intracellular bio-distribution of drugs delivered by upconversion nanoparticles^34^,^35^.

## Methods

### Preparation of upconversion nanocrystals

Lanthanide-doped nanorods and nanoparticles were prepared through a hydrothermal method^23^ and a coprecipitation method^36^, respectively. The as-prepared nanocrystals were washed with HCl to remove oleic acid molecules that were used as surface-capping ligands during the synthesis. Detailed experimental procedures are provided in the Supplementary Methods.

### Preparation of cells

Human cervical carcinoma cells were seeded onto 100-nm-thick silicon-nitride membranes at a density of 9,000 cells cm^−2^ in Dulbecco's Modified Eagle's medium containing fetal bovine serum (10%), penicillin (100 units ml^−1^) and streptomycin (100 μg ml^−1^). After 24 h and a brief wash with phosphate-buffered saline, the cells were incubated in complete medium containing the as-synthesized NaYF_4_:Yb/Tm (60/2%) nanoparticles (10 μg ml^−1^) for another 24 h. Following another wash with Hepes-buffered saline, the particle-treated cells were then transferred to a solution of 2% glutaraldehyde and stored overnight prior to intermediate dehydration using an increasing ethanol gradient. Complete dehydration was then achieved by critical-point drying.

### Instrumentation and imaging

Photoluminescence images were taken by an Olympus BX51 optical microscope equipped with a 980-nm diode laser. The α-particle-induced luminescence imaging was achieved by collecting the luminescence photons with a customized double-piece parabolic mirror. The collected photons were then detected by a Hamamatsu photomultiplier tube (PMT) R7400P equipped with the photon counting unit C9744. The data were collected and processed using the IONDAQ data acquisition system^37^ to generate the ionoluminescence images (Supplementary Fig. 13). The energy loss of an ion transmitted through a sample depends on the sample composition and thickness. Thus, the areal density can be expressed by equation (1):


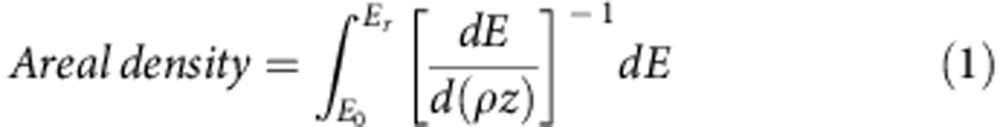


where *E*_*0*_ is the initial ion energy, *E*_*r*_ is the remaining energy of the ion after passing through the sample, and *ρ*=*ρ*(*z*) is the mass density of the sample at a depth of *z*. Through scanning transmission ion microscopy, the transmitted ion energies and number of ions at each pixel within the scanned area can be measured by a Si surface barrier detector to render the areal density map.

## Additional information

**How to cite this article:** Mi, Z. *et al*. Subwavelength imaging through ion-beam-induced upconversion. *Nat. Commun.* 6:8832 doi: 10.1038/ncomms9832 (2015).

## Supplementary Material

Supplementary Information Supplementary Figures 1-13, Supplementary Table 1, Supplementary Notes, Supplementary Methods and Supplementary References.
