# Supplementary material for: Subwavelength imaging through ion-beam-induced upconversion
Source: Nat Commun. 2015 Nov 12;6:8832. doi: 10.1038/ncomms9832 (PMC4660043; doi:10.1038/ncomms9832)
Supplement: Supplementary Information — Supplementary Figures 1-13, Supplementary Table 1, Supplementary Notes, Supplementary Methods and Supplementary References. [file ncomms9832-s1.pdf]

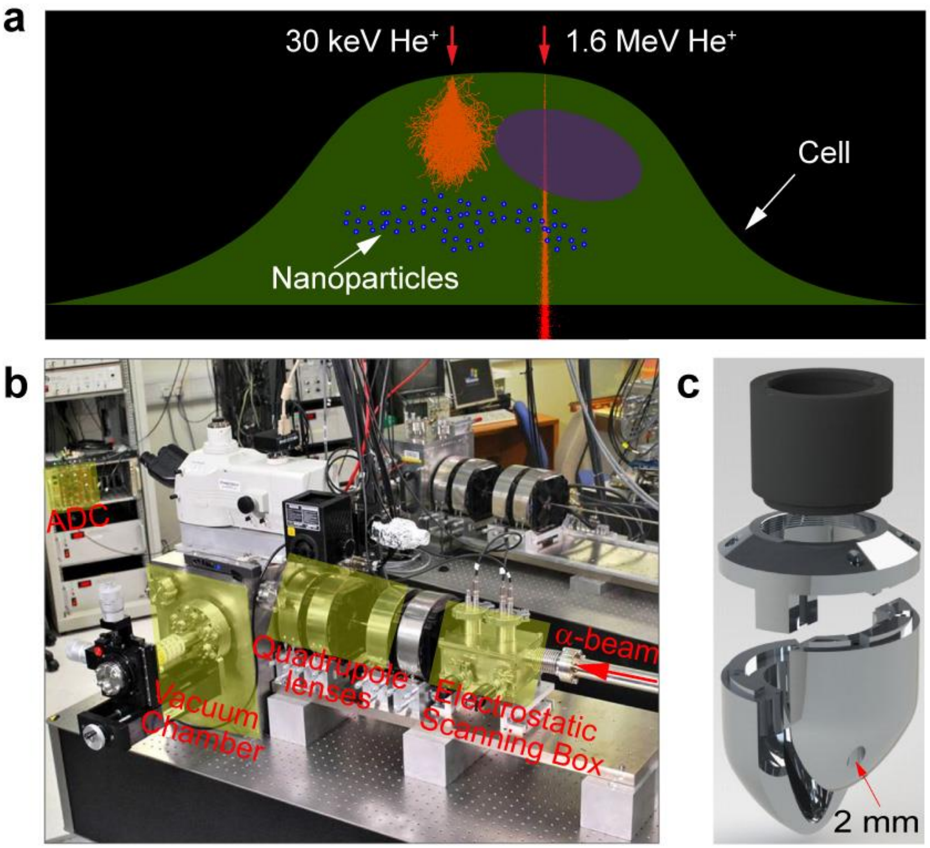

Supplementary Figure 1: Basic instrumentation setup for ionoluminescence imaging. (**a**) Schematic showing the different penetration ability of slow (30 keV) and fast (1.6 MeV) alpha particles. (**b**) Photograph showing the end-station of the ion beam imaging facility. Briefly, the accelerator-produced MeV α-beam was focused down to the sample situated in a vacuum chamber by using a spaced triplet of magnetic quadrupole lenses and scanned over the sample through electrostatic deflection. The signals produced during ion-sample interaction were subsequently collected and processed by analog-to-digital converters (ADC). (**c**) Customized design of a double-piece parabolic mirror for the collection of luminescence photons. The 2-mm hole (marked by the red arrow) is designed to allow the entry and exit of the ion beam.

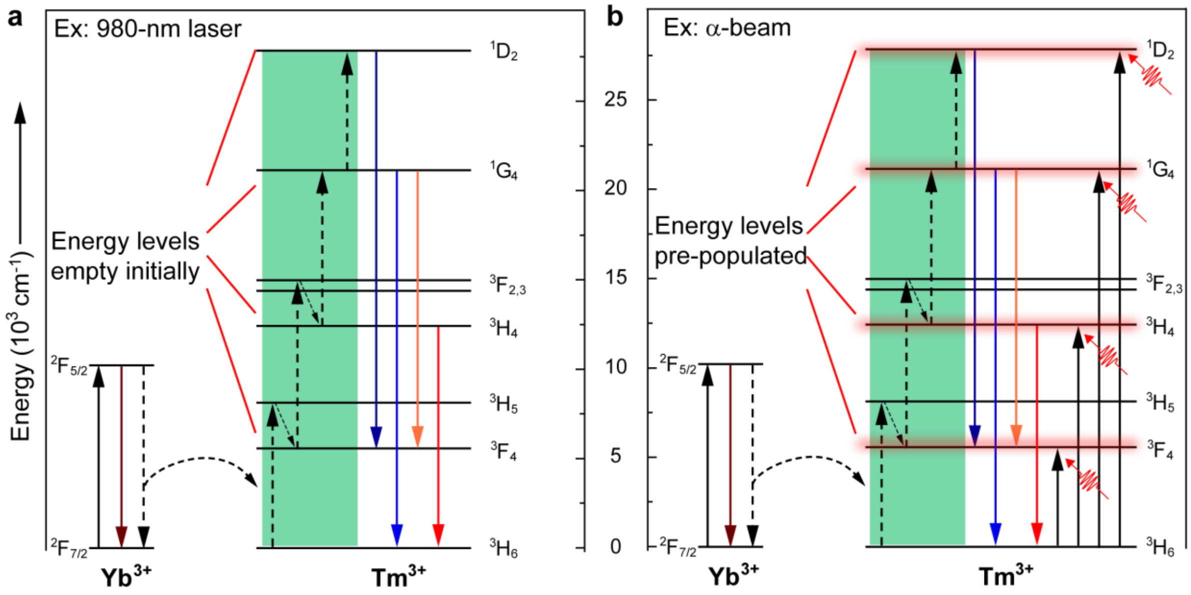

Supplementary Figure 2: Proposed upconversion mechanisms under 980-nm laser and ion-beam stimulation. (**a**) Energy diagram showing a typical energy transfer upconversion (ETU) process in $NaYF_4$:Yb/Tm crystal when excited by a 980-nm diode laser. ETU features a ladder-like process in which energy levels are populated and accumulated from the lowest to the highest. (**b**) Energy diagram showing the energy transfer process in $NaYF_4$:Yb/Tm crystal when excited by an α-beam. Unlike the interaction between the 980-nm laser and the crystal in the ETU process, the interaction between the α-beam and the crystal lattice generates a large number of excitons, carrying energies ranging from 0.1 eV to 1000 eV. Such energies not only excite a large number of Yb^{3+} but also allow a substantial amount of Tm^{3+} to be populated prior to the energy transfer from Yb^{3+} to Tm^{3+}, thereby facilitating the photon upconversion process.

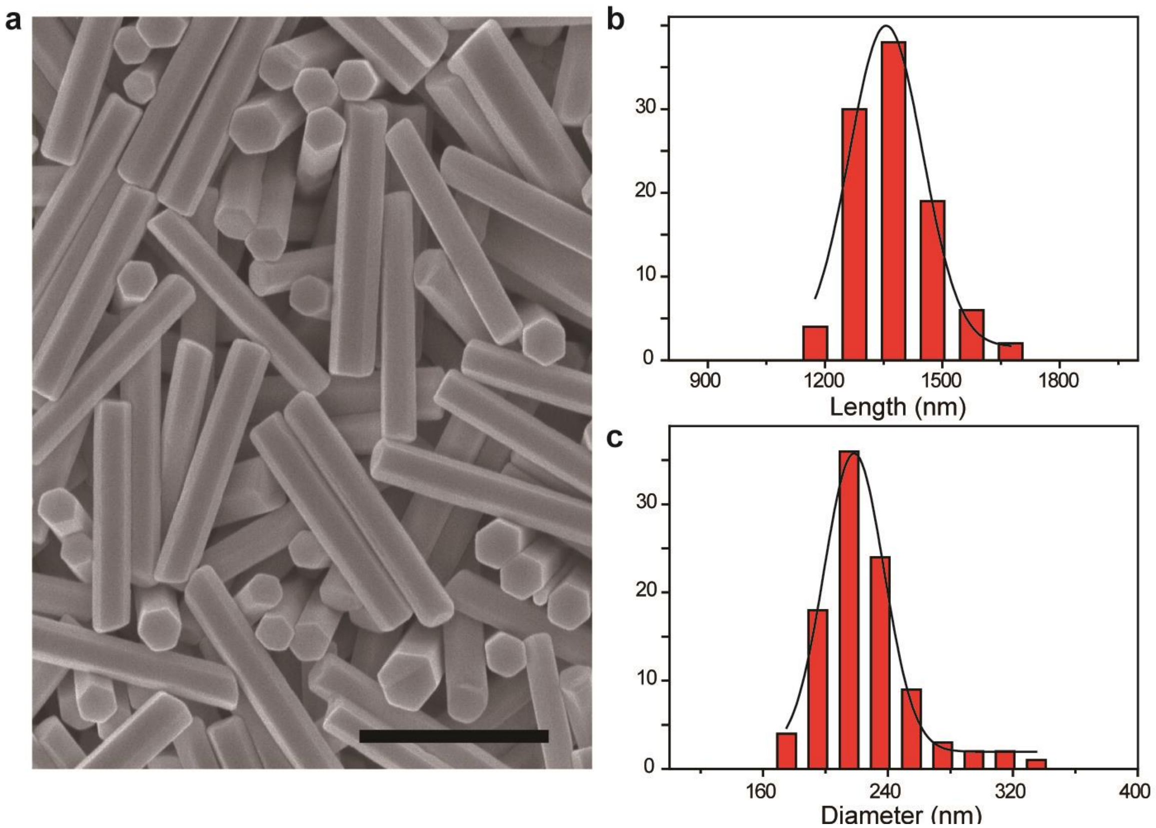

Supplementary Figure 3: Scanning electron microscopic analysis. (**a**) A typical scanning electron microscopic image of $NaYF_4$:Yb/Tm(60/2 mol%) crystals featuring a hexagonal-prism morphology. Scale bar is 1 μm. (**b**) Length distribution histogram of the as-synthesized $NaYF_4$:Yb/Tm(60/2 mol%) crystals, showing an average length of 1.4 μm. (**c**) Diameter distribution histogram of the as-synthesized $NaYF_4$:Yb/Tm(60/2 mol%) crystals, showing an average diameter of 220 nm. Note that the diameter and length of the crystals can be varied by adjusting Yb^{3+} doping concentration.

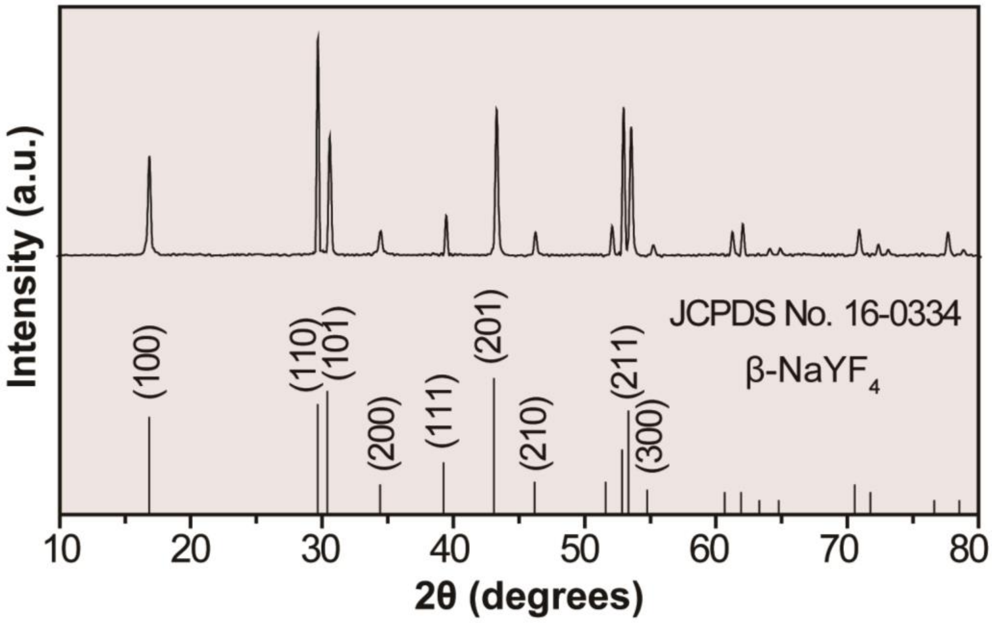

Supplementary Figure 4: X-ray diffraction characterization. All diffraction peaks from the as-synthesized $NaYF_4$:Yb/Tm(60/2 mol%) nanorods can be well indexed in accordance with hexagonal-phase $NaYF_4$ (β-$NaYF_4$) crystal structure (Joint Committee on Powder Diffraction Standards file No. 16-0334).

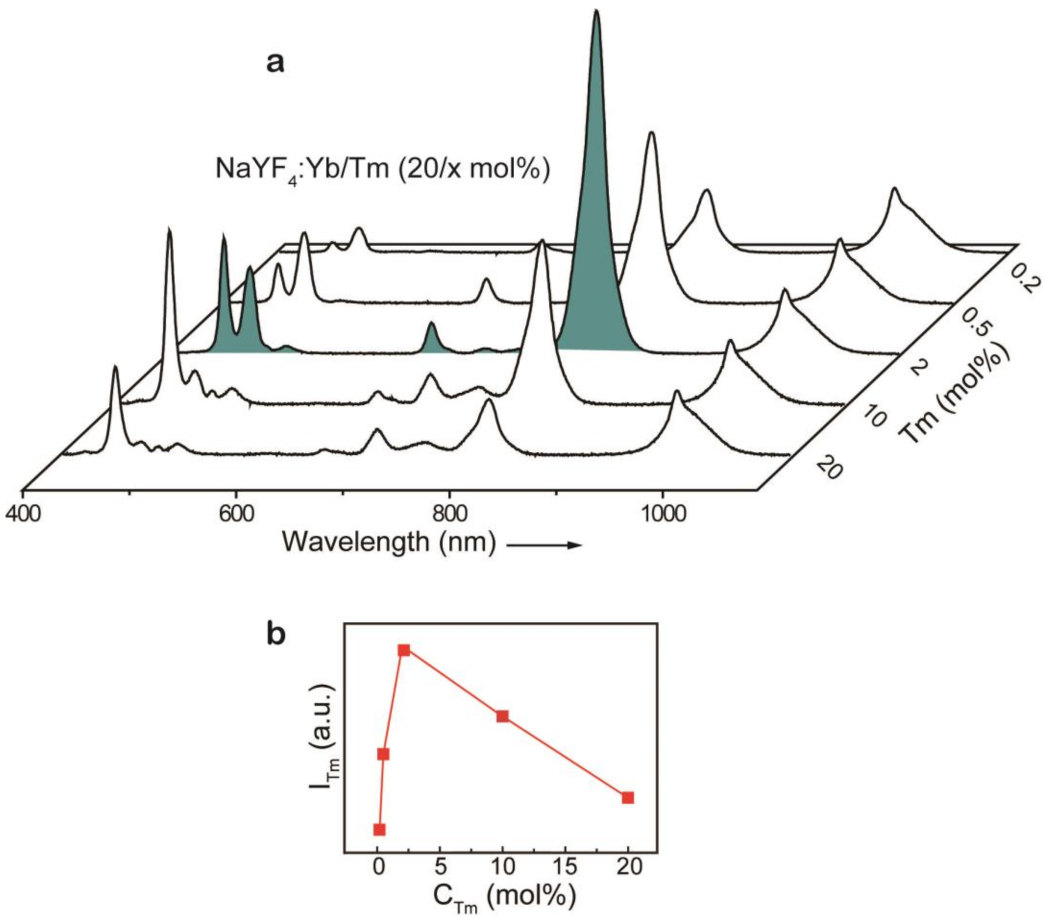

Supplementary Figure 5: Spectroscopic characterization of $NaYF_4$:Yb/Tm (20/x mol%) crystals. (**a**) Emission spectra of the crystals excited by 1.6 MeV α-particles. (**b**) Integrated intensities of Tm^{3+} emission plotted against doping concentration, showing that 2 mol% is the optimal doping concentration for maximal emission output in the Vis-NIR range (400-900 nm). Note that the concentration of Yb^{3+} is kept constant at 20 mol%.

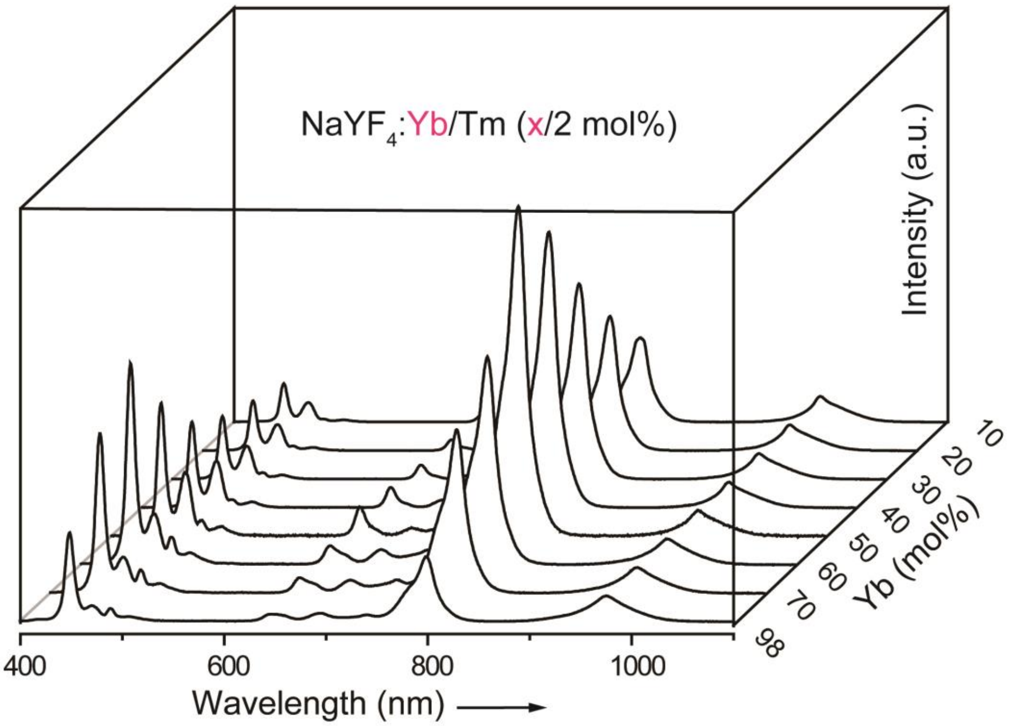

Supplementary Figure 6: Spectroscopic analysis of $NaYF_4$:Yb/Tm (x/2 mol%) crystals. Emission spectra of the crystals recorded with various Yb^{3+} doping concentrations (10-98 mol%) under the excitation of 1.6 MeV α-particles.

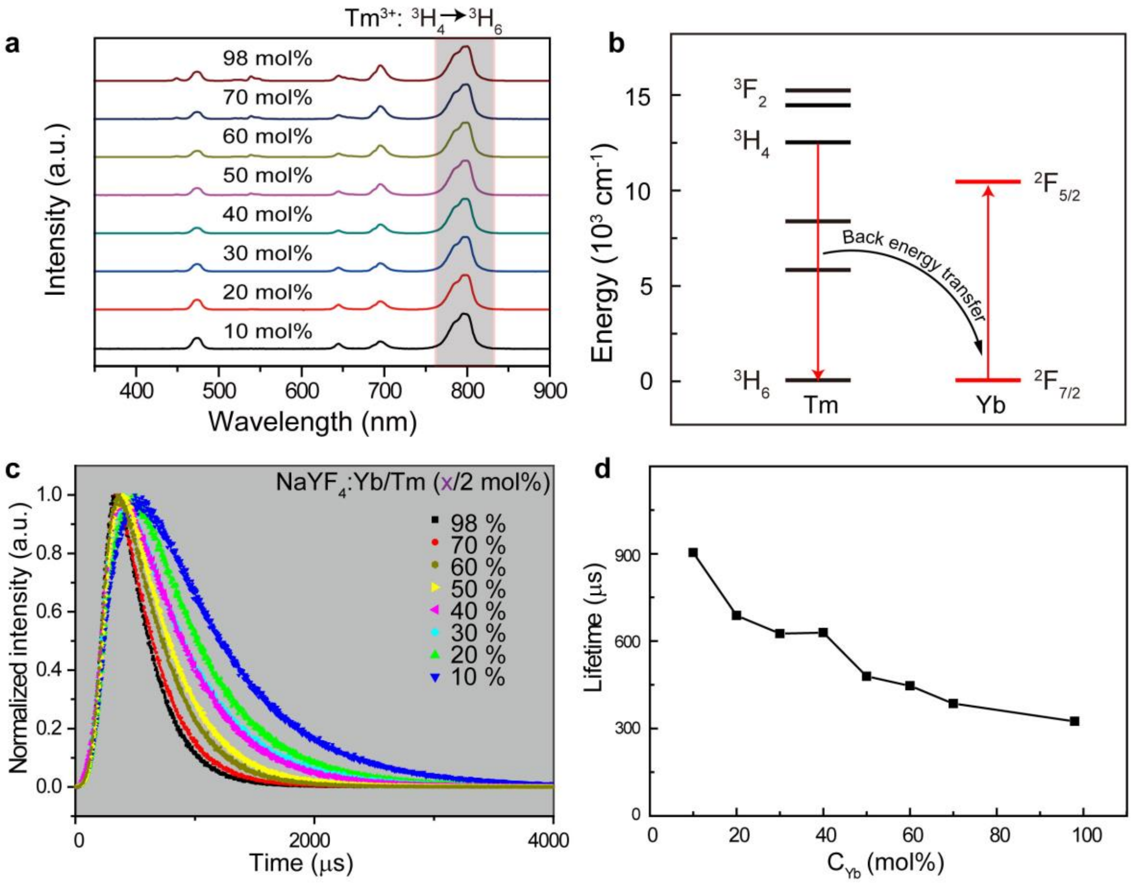

Supplementary Figure 7: Characterization of back-energy-transfer process in $NaYF_4$:Yb/Tm crystal. (**a**) Room-temperature upconversion emission spectra of $NaYF_4$:Yb/Tm (x/2 mol%) crystals when excited with a 980-nm diode laser at a power density of ~ 100 W cm^{-2}. The highlighted emission bands originate from the optical transition $^3H_4 \rightarrow {}^3H_6$ of Tm^{3+}. (**b**) Energy diagram showing the back-energy-transfer process from Tm^{3+} to Yb^{3+}. Although the energy mismatch (ΔE = 2650 cm^{-1}) involved in this transfer is very large, the back-energy-transfer efficiency can be enhanced by increasing Yb^{3+} concentration to provide a shortened distance between Tm^{3+} and Yb^{3+}. (**c**) Upconversion luminescence decay curves of the emission bands at 800 nm (Tm^{3+}: $^3H_4 \rightarrow {}^3H_6$) recorded from $NaYF_4$:Yb/Tm (x/2 mol%)(x = 10, 20, 30, 40, 50, 60, 70, and 98) crystals under 980-nm laser excitation. (**d**) Lifetimes of Tm^{3+} at its $^3H_4$ level plotted against Yb^{3+} doping concentration, showing a prominent decrease in lifetime with increasing Yb^{3+} doping concentration. The results observed under 980-nm laser excitation suggest that a back-energy-transfer process in $NaYF_4$:Yb/Tm crystals is likely to occur with α-particles as the excitation source.

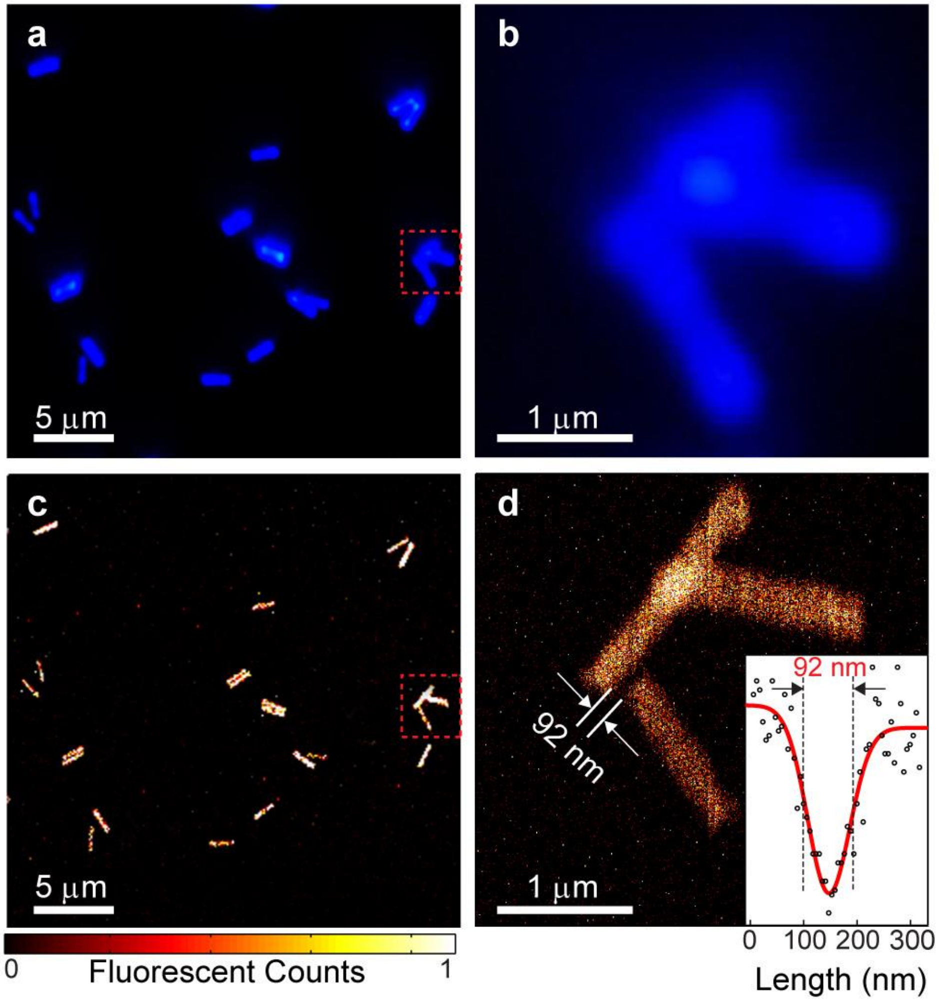

Supplementary Figure 8: Spatial resolution determination by ionoluminescence microscopy. (**a**) Upconversion photoluminescence imaging of $NaYF_4$:Yb/Tm (60/2 mol%) crystals when irradiated with a 980 nm laser. (**b**) An enlarged imaging area marked with a dotted square in **a**. The blurring of boundaries between the crystal and the background is due to the optical diffraction limit, estimated to be 300 nm in this study. (**c**) Ionoluminescence imaging of $NaYF_4$:Yb/Tm (60/2 mol%) crystals under α-beam irradiation. (**d**) An enlarged imaging area (marked with a dotted square **c**) shows that it is possible to measure a sub-100 nm gap between two adjacent upconversion crystals by the α-beam microscopy.

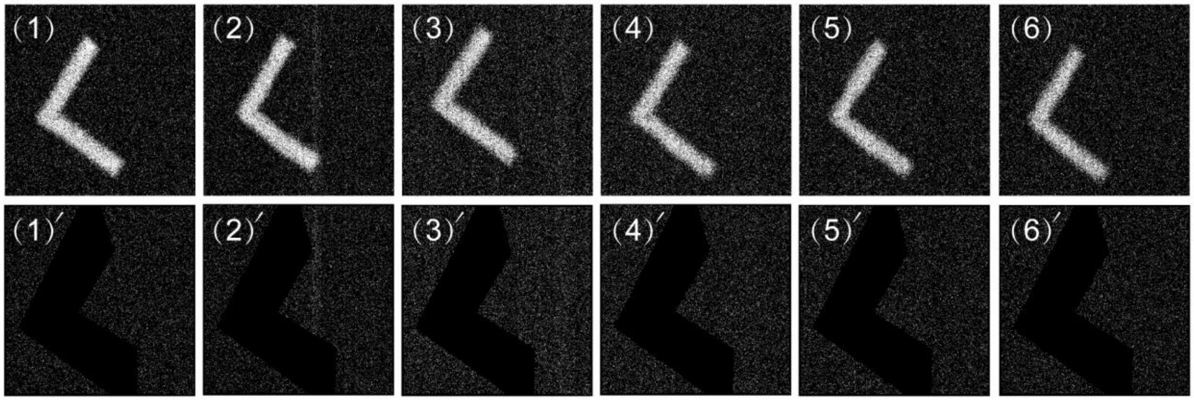

Supplementary Figure 9: Ionoluminescence intensity measurements. The strategy for extracting the ionoluminescence counts from the image of $NaYF_4$:Yb/Tm (60/2 mol%) nanorods to quantify iono-bleaching under 1.6 MeV α-particle irradiation. First row (panels 1-6): Images of two nanorods after receiving a He^+ dosage of 1.46×10^{14}, 2.93×10^{14}, 4.39×10^{14}, 5.86×10^{14}, 7.32×10^{14} and 8.79×10^{14} ions/cm^2, respectively. Total luminescence counts from each of these six images can be obtained. Second row (panels 1'-6'): Reconstructed images corresponding to the images in the first row after deducting the ionoluminescence counts of the two nanorods. The luminescence counts in images (panels 1'-6') come from the substrate and the detection noise, referring to the background counts. By averaging total counts in each image of the second row, averaged background counts per pixel can be obtained. Therefore, the total background counts in each image of the first row can be estimated. Finally, net ionoluminescence counts from the two nanorods can be calculated by subtracting the background counts in each image of the first row.

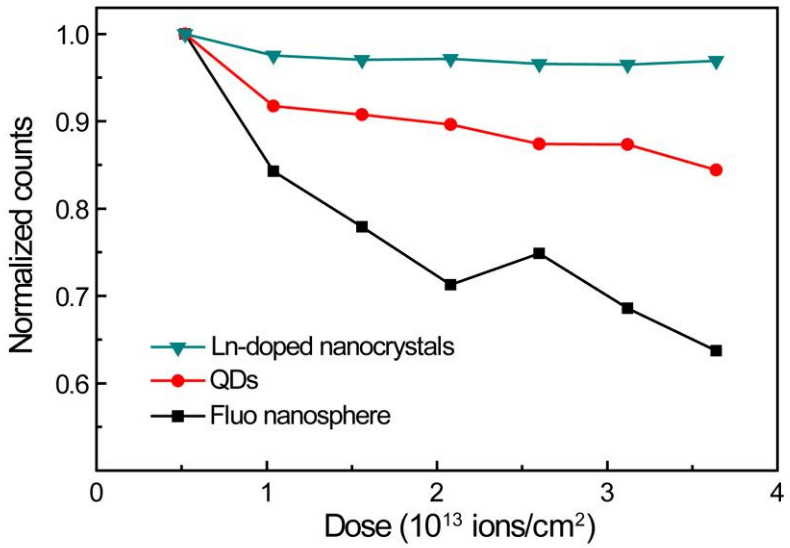

Supplementary Figure 10: Iono-bleaching analysis. Iono-bleaching analysis for organic-based probes (Fluo nanosphere, Thermo Fisher Scientific Inc), CdSe@ZnS quantum dots (QDs, homemade), and $NaYF_4$:Yb/Tm (60/2 mol%) nanoparticles. Upon continuous ion exposure at 3.6×10^{13} ions/cm^2, 16% and 37% of the luminescence signal were bleached for quantum dots and organic probes, respectively. In stark contrast, only 3% of the luminescence signal was bleached for $NaYF_4$:Yb/Tm (60/2 mol%) nanoparticles. These results show that lanthanide-doped materials have a much higher resistance to iono-bleaching than quantum dots or organic materials.

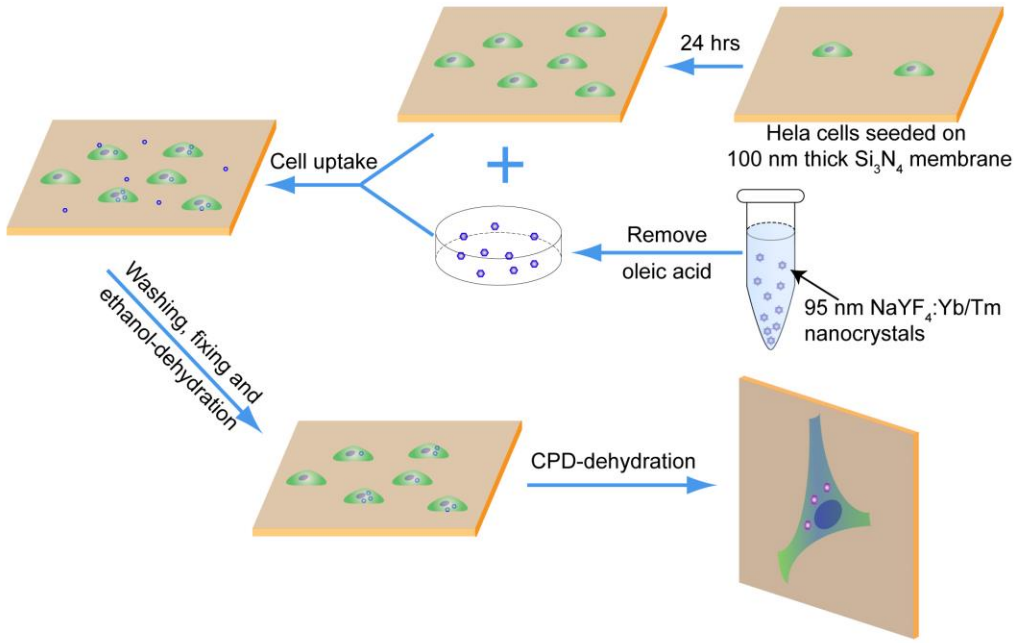

Supplementary Figure 11: Experimental procedure for particle-internalization by HeLa cells. Schematic representation of the procedures used for preparing $NaYF_4$:Yb/Tm (60/2 mol%) nanoparticle-tagged HeLa cells for ionoluminescence imaging. The HeLa cells were seeded onto 100-nm thick silicon nitride (Si_3N_4) membranes and incubated overnight (24 hours). The oleic acid was removed from the as-synthesized $NaYF_4$:Yb/Tm(60/2 mol%) nanoparticles, and the ligand-free nanoparticles were then used for cell uptake. The cells deposited on the Si_3N_4 membrane were incubated in complete medium containing $NaYF_4$:Yb/Tm(60/2 mol%) nanoparticles (10 μg/ml) for another 24 hours, followed by washing to remove surplus nanoparticles, cell fixing, and ethanol-dehydration. Complete dehydration was achieved by critical point drying (CPD).

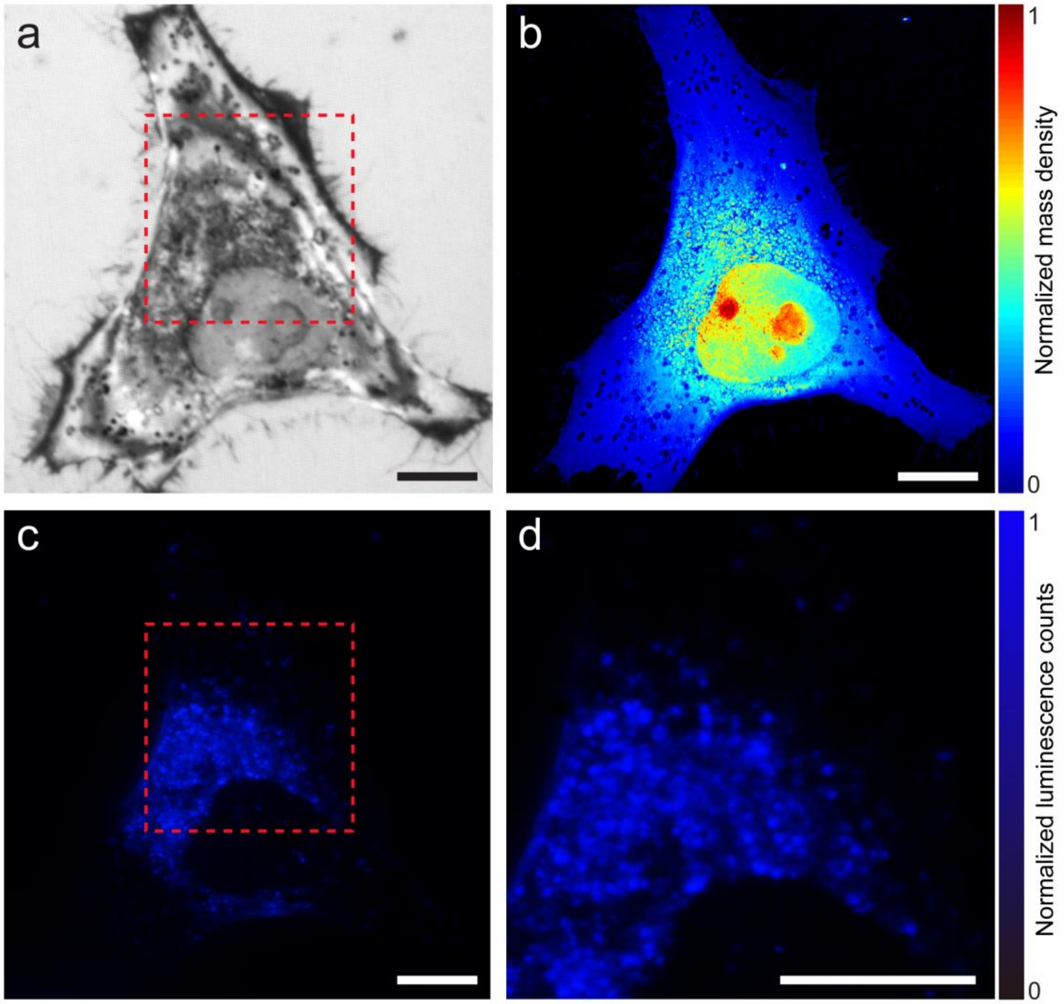

Supplementary Figure 12: Single-cell imaging analysis. (**a**) Optical image of a whole HeLa cell taken using a 100 x objective lens. (**b**) Scanning transmission ion microscopy image showing cellular structures. (**c**) Whole-cell photoluminescence image showing the particle emission under 980 nm laser excitation. (**d**) Enlarged photoluminescence image extracted from the area marked with a dotted square in **c**. Scale bars in the four images are all 10 μm.

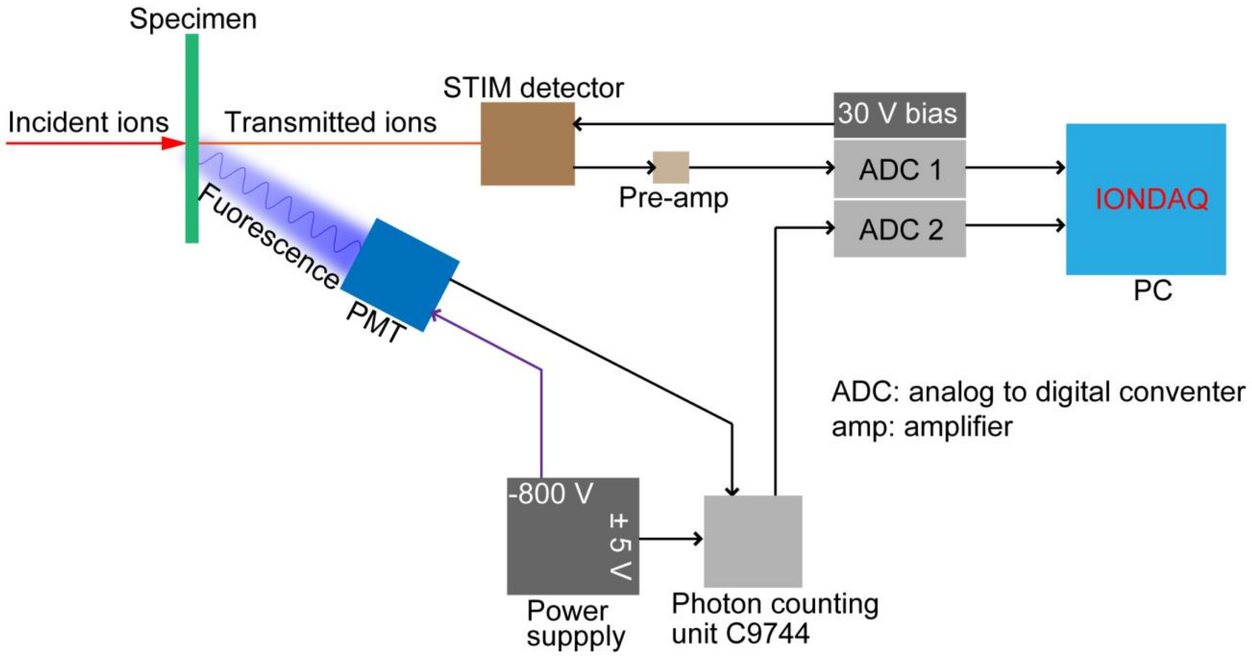

Supplementary Figure 13: Signal detection and processing. Schematic showing signal detection and processing for both ionoluminescence and STIM imaging. For ionoluminescence imaging, the photon counting unit C9744 is used in combination with the PMT for counting the photons. The signal output from the C9744 unit is processed by an analog to digital converter (ADC 2). For STIM, the signal output from the surface barrier detector is first pre-amplified, and then converted to digitals by ADC 1. The IONDAQ data acquisition system is finally employed to collect and further process the digital signals obtained from ADCs.

Supplementary Table 1: Comparison of 1.6 MeV α-beam and 980 nm diode laser as the excitation sources for $NaYF_4$:Yb/Tm nanocrystals.

Excitation source	Beam power (W/cm^2)	Yb^{3+} absorption cross-section(cm^{-2})	Imaging resolution (nm)	Excitation-emission mechanism
1.6 MeV α-beam	~10^2	3.2×10^{-16}*	28	Both ground state absorption (GSA) and energy transfer upconversion (ETU) processes contribute to the emission of Tm^{3+}; Pre-population of the excited states of Tm^{3+} facilities the energy transfer upconversion.
980 nm diode laser	~10^2	9.1×10^{-21}**	253	Only Yb ions can absorb the pumping photons; the ladder-like upconversion processes must be stepwise.

*Calculated using eq. 9. **This value was found in refs.1, 8 and 9.

Supplementary Notes

Calculation of ion beam power. The power per cm[2] (P) of a 1.6 MeV helium ion beam focused down to a spot size of 30 × 30 nm[2] can be calculated as

$$\mathrm{P}=\frac{E_0 R}{A}=\frac{1.6MeV\times 20000/s}{(30nm)^2}=5.7\times 10^2 W\cdot cm^{-2} \quad (1)$$

where E_0 (1.6 MeV) is the energy of an incident helium ion, A (30 × 30 nm^2) is the beam spot size, and R (20000/s) is the counting rate of exposed ions.

It should be noted that the power level (10^2 W cm^{-2}) of the ion beam under investigation is comparable to the power range (1-10^3 W cm^{-2}), typically employed to induce an upconversion process in $NaYF_4$:Yb/Tm nanocrystals using a 980 nm diode laser.

Regardless of the power, merits of the ion beam as the excitation source include two aspects. First, it can drive both downconversion and upconversion luminescence to occur simultaneously due to the broad energy distribution of ionized electrons. Second, under the ion beam excitation, the absorption cross-section of Yb^{3+} is much bigger compared to that of the 980 nm diode laser excitation.

Energy spectrum of first-generation ionized electrons. The ionization due to the interaction of MeV helium ions with the $NaYF_4$:Yb/Tm crystals results in the ejection of ionized electrons (or so called δ-rays) and the production of 'electron holes' inside the crystals. The cross-section of these δ-rays can be dealt with the Hansen-Kocbach-Stolterfoht (HKS) model[1,6]. In the HKS model, single differential cross-section (SDCS) for the emission of a δ-ray with energy between ε and $\varepsilon+d\varepsilon$ from the ith shell of an atom is written as

$$\frac{d\sigma_{\mathrm{i}}(\varepsilon)}{d\varepsilon}=\frac{4a_0^2 Z_1^2}{3\pi R v_1^2 \alpha k_c^3 \hat{k}_t}\Bigg[3\arctan(\hat{K}_m+\hat{k}_t)-3\arctan(\hat{K}_m-\hat{k}_t) +\frac{5(\hat{K}_m+\hat{k}_t)+3(\hat{K}_m+\hat{k}_t)^3}{[1+(\hat{K}_m+\hat{k}_t)^2]^2}-\frac{5(\hat{K}_m-\hat{k}_t)+3(\hat{K}_m-\hat{k}_t)^3}{[1+(\hat{K}_m+\hat{k}_t)^2]^2}\Bigg] \quad (2)$$

where

$a_0=5.3\times 10^{-7}\,\mathrm{cm}$	Bohr radius
$Z_1=2$	atomic number of impinging particle
$R=13.6\ \mathrm{eV}$	Rydberg energy
$v_1=(T/R)^{1/2}$	reduced velocity of the impinging particle
$T=(E/Z_1)(m_e/m_{He})=E/7344Z_1$	reduced kinetic energy

$E = 1.6\times10^{6}\text{eV}$	energy of an incident ion
B_i	binding energy of the ith shell electron of an atom
$\alpha = (B_i / R)^{1/2}$	mean initial momentum
$k = (\varepsilon / R)^{1/2}$	momentum of outgoing δ-ray
$\alpha_c = \alpha(1+0.7\frac{v_1^2}{v_1^2+k^2})$	small modification to α
$K_m = (\alpha^2 + k^2) / 2v_1$	minimum momentum transfer
$k_c = (k^2 + \frac{2\alpha^2}{\ln(2v_1^2/\alpha^2)})^{1/2}$	
$k_t = (k^2 + 0.2\alpha^2\sqrt{\frac{v_1}{\alpha}})^{1/2}$	
$\hat{k}_t = k_t / \alpha_c$	
$\hat{K}_m = K_m / \alpha_c$	

And the SDCS, $\frac{d\sigma_i(\varepsilon)}{d\varepsilon}$, which has the unit of cm^2/eV, represents the probability of ionization of an ith shell electron with the energy between ε and $\varepsilon+d\varepsilon$ per unit energy (in eV). The total SDCS for the emission of a δ-ray with energy between ε and $\varepsilon+d\varepsilon$ in the $NaYF_4$:Yb/Tm crystal is

$$\sigma_\varepsilon = \sum_i n_i \frac{d\sigma_i}{d\varepsilon} \tag{3}$$

where n_i is the weight function of an ith shell electron.

The analytical calculation result for the crystal $NaYF_4$:Yb/Tm(60/2%) is shown in Fig. 1b in the main text.

Derivation of absorption cross-section of Yb^{3+} from ionized electrons. In a series of $NaY_{0.98-x}Tm_{0.02}Yb_xF_4$ nanocrystals with fixed Tm^{3+} concentration and variable Yb^{3+} concentration, we make three assumptions in order to simplify the calculation for the absorption cross-section:

(1) Considering only the ionization while neglecting the other energy deposition processes;

(2) Neglecting the absorption of Tm^{3+} ions while assuming only Yb^{3+} ions have the absorption since Tm^{3+} concentration is low;

(3) Eliminating the effect of phonon (heat) production during the absorption processes.

Let σ denote the average absorption cross-section of Yb^{3+} and t the thickness of the nanocrystal along the beam propagation path. Then the absorption probability of Yb^{3+} ions from the ionized electrons (probably by absorption of the energy released after electron-hole recombination) with energy ε is defined as

$$\sigma t \cdot N_{\varepsilon}^{e^-}$$

where $N_{\varepsilon}^{e^-}$ is the number density of ionized electrons with energy ε, and

$$N_{\varepsilon}^{e^-} = \frac{\sigma_{\varepsilon} \cdot \Delta\varepsilon \cdot N^{e^-}}{\int_0^{\varepsilon_m} \sigma_{\varepsilon} d\varepsilon} \quad (4)$$

where σ_{ε} is the single differential cross-section (SDCS) for the emission of a δ-ray with energy between ε and $\varepsilon + d\varepsilon$ in the crystal, which can be calculated using eq. 3; ε_m is the maximum energy of the δ-rays; $\Delta\varepsilon$ is a small energy interval chosen for the numerical calculation; N^{e^-} is the number density of δ-rays.

The effective number of electrons contributed by Na^+, Y^{3+}, Tm^{3+}, Yb^{3+}, and F^- is 9, 26, 34, 33 and 9 respectively, considering the fact that binding energy of an atomic electron to be ionized cannot exceed the energy of an incident helium ion (1.6 MeV)[1,7]. Thus, the number density of ionized electrons can be derived as follows:

$$N^{e^-} = [9 + 26 \times (0.98 - x) + 34 \times 0.02 + 33x + 9 \times 4]N = (71.2 + 7x)N \quad (5)$$

where N is the number density of $NaY_{0.98-x}Tm_{0.02}Yb_xF_4$ crystals with a mass density of ρ and a molar mass of M, and

$$N = \frac{\rho N_A}{M} \quad (6)$$

$$M = 193.5 + 84x \quad (7)$$

Equation for total absorption energy can be written as

$$\int_0^{\varepsilon_m} \sigma t N_{\varepsilon}^{e^-} \cdot N_{\varepsilon}^{e^-} d\varepsilon = \int_{\varepsilon_0}^{\varepsilon_m} N_{\varepsilon}^{e^-} d\varepsilon \quad (8)$$

where ε_0 is the minimum energy absorbable by Yb^{3+} ions, i.e. the energy difference between $^2F_{5/2}$ and $^2F_{7/2}$ levels of Yb^{3+}.

By substituting eqs. 4-7 into eq. 8, one can derive

$$\sigma = \frac{\int_0^{\varepsilon_m} \sigma_{\varepsilon} d\varepsilon \cdot \int_{\varepsilon_0}^{\varepsilon_m} \sigma_{\varepsilon} d\varepsilon}{\Delta\varepsilon \cdot \int_0^{\varepsilon_m} \sigma_{\varepsilon}^2 d\varepsilon} \frac{193.5 + 84x}{t\rho N_A (71.2 + 7x)} \quad (9)$$

Using the parameters $\Delta\varepsilon$ (0.001 eV), t (150 nm), and ρ (4.21 g cm^{-3}), we can calculate the average absorption cross-section of Yb^{3+} numerically, as shown in Supplementary Table 1.

Supplementary Methods

Materials. Yttrium(III) nitrate (99.9%), ytterbium(III) nitrate (99.9%), thulium(III) nitrate (99.9%), erbium(III) nitrate (99.9%), yttrium(III) acetate hydrate (99.9%), ytterbium(III) acetate hydrate (99.9%), thulium acetate hydrate (99.9%), sodium hydroxide (NaOH, >98%), ammonium fluoride (NH_4F, >98%), oleic acid (90%) were all purchased from Sigma-Aldrich and used as received.

Synthesis of $NaYF_4$:Yb/Tm nanorods. The $NaYF_4$ nanorods (~ 2 μm in length) were synthesized by a hydrothermal reaction[1]. In a typical experiment, NaOH (0.3 g; 7.5 mmol) was first dissolved in 1.5 mL of DI water, followed by addition of 5 mL of oleic acid and 5 mL of ethanol while stirring. Thereafter, an aqueous solution of NH_4F (2 M; 1 mL) was added to yield a turbid mixture. Subsequently, an aqueous solution (2 mL) containing $Y(NO_3)_3$ (0.352 mmol), $Yb(NO_3)_3$ (0.04 mmol) and $Tm(NO_3)_3$ (0.008 mmol) was added into the mixture and kept stirring for 20 min. The resulting mixture was then transferred to a 20-mL Teflon-lined autoclave and heated at 220 °C for 12 h. After cooling down to room temperature, the reaction product was isolated by centrifugation and washed with ethanol. A series of $NaYF_4$ nanorods with varied Yb^{3+} doping concentrations (10-98 mol%) were synthesized following a similar procedure as above, except for the molar amounts of loaded lanthanide ions.

Synthesis of $NaYF_4$:Yb/Tm (60/2 mol%) nanoparticles. The $NaYF_4$ nanoparticles (~ 95 nm in diameter) were synthesized by a co-precipitation method[2]. In a typical experiment, 3 mL of oleic acid and 7 mL of ODE were mixed with an aqueous solution (2 mL) of YCl_3 (0.152 mmol), $YbCl_3$ (0.24 mmol), and $TmCl_3$ (0.008 mmol) in a 50 mL round-bottom flask under vigorous stirring. The resulting mixture was then heated at 150 °C for 1 h to yield a transparent solution of lanthanide-oleate complex. After cooling down to room temperature, the mixture was added into a methanol solution (6 mL) containing NH_4F (1.6 mmol) and NaOH (1 mmol), followed by the removal of methanol at 100 °C under stirring. The resultant mixture was then heated to 300 °C and kept for 2 h under a nitrogen atmosphere. The nanoparticles were precipitated by addition of ethanol and collected by centrifugation.

Preparation of ligand-free $NaYF_4$:Yb/Tm nanocrystals. The ligand-free nanocrystals were prepared through an acid-washing procedure[3]. Typically, 10 mg as-prepared $NaYF_4$:Yb/Tm nanocrystals were dispersed in a mixture containing 1 mL ethanol and 1 mL aqueous solution of HCl (2 M). The dispersion was ultrasonicated for 15 min to thoroughly remove those hydrophobic oleic acid molecules tethered on the surface of nanocrystals. The ligand-free nanocrystals were washed with ethanol, collected by centrifugation, and re-dispersed in water. For single nanorod imaging purpose, the aqueous dispersion of $NaYF_4$:Yb/Tm nanorods was diluted 1000 times. One drop of the diluted dispersion was gently cast on

the surface of a Si_3N_4 substrate with thin windows (100 nm in thickness), followed by slow evaporation at room temperature. The dried substrate was then mounted on our home-made ion-beam microscope for image construction. On a separate note, the undiluted solution was used for casting on substrate to impose a pronounced optical signal for spectra measurement purpose.

Physical Measurements. Those as-synthesized nanocrystals, including nanorods and nanoparticles, were examined under a transmission electron microscopy (TEM, JEM-2100F) operating at an acceleration voltage of 200 kV and a scanning electron microscopy (SEM, JEOL JSM-6701F) working at an acceleration voltage of 5 kV. Powder X-ray diffraction (XRD) data were recorded on a Siemens D5005 X-ray diffractometer with Cu Kα radiation (λ = 1.5406 Å). Upconversion photoluminescence spectra were obtained through the use of a spectrometer (QE65000, Ocean Optics) equipped with a 980-nm continuous-wave (CW) diode laser as the excitation source. Upconversion photoluminescence imaging was performed using an upright Olympus BX51 microscope coupled with a 980-nm diode laser through fibre output. The photoluminescence micrographs were recorded with a Nikon DS-Ri1 imaging system.

The 1.6 MeV helium ion beam is produced by a High Voltage Engineering Europa Singletron™ ion accelerator. The beam focus is achieved using a spaced triplet of compact Oxford Microbeams OM52 magnetic quadrupole lenses, while the beam scanning over the sample is realized through electrostatic deflection. Ionoluminescence of the sample is collected by a customized double-piece parabolic mirror[4]. Two holes (2 mm in diameter) are designed in each piece of the parabolic mirror to allow entering and exiting of the ion beam. The light emission is guided out of a vacuum chamber (10^{-6} mbar) by a fibre through an optical feedthrough. Luminescent photons are then captured and detected, either by the Hamamatsu photomultiplier tube R7400P for alpha-particle-induced luminescence imaging or by the spectrometer (QE65000) for spectral measurement. The photon counting unit C9744 is used together with the PMT for counting the photons detected (Supplementary Figure 13). The signal output from the C9744 unit is subsequently processed by an analog to digital converter (ADC) before the acquisition by IONDAQ program[5] for imaging. After interaction with the sample, directly transmitted ions are detectable by a silicon detector (surface barrier, ORTEC®), located along the axis of the beam behind the sample and used for scanning transmission ion microscopy (STIM) imaging of the sample. The signal output from the surface barrier detector is pre-amplified in case of being buried in the noise during the transmission. The pre-amplified signal is then converted to digitals by ADC 1 and inputted into IONDAQ for STIM imaging.

Supplementary References

1. Zhang, Y. *et al.* Multicolor barcoding in a single upconversion crystal. *J. Am. Chem. Soc.* **136**, 4893-4896 (2014).

2. Wang, F., Deng, R. & Liu, X. Preparation of core-shell $NaGdF_4$ nanoparticles doped with luminescent lanthanide ions to be used as upconversion-based probes. *Nature Protoc.* **9**, 1634-1644 (2014).

3. Wang, F. *et al.* Tuning upconversion through energy migration in core–shell nanoparticles. *Nature Mater.* **10**, 968-973 (2011).

4. Vanga, S. K. *et al.* Development of a new light collection and detection system optimized for ion beam induced fluorescence microscopy. *Nucl. Instrum. Methods Phys. Res. B.* **348**, 111-114 (2015).

5. Bettiol, A. A., Udalagama, C., & Watt, F. A new data acquisition and imaging system for nuclear microscopy based on a Field Programmable Gate Array card. *Nucl. Instrum. Methods Phys. Res. B.* **267**, 2069-2072 (2009).

6. Udalagama, C., Bettiol, A. A., & Watt, F. Stochastic spatial energy deposition profiles for MeV protons and keV electrons. *Phys. Rev. B* **80**, 224107 (2009).

7. NIST atomic spectra database ionization energies form. Available at: http://physics.nist.gov/PhysRefData/ASD/ionEnergy.html

8. Xie, X. *et al.* Mechanistic investigation of photon upconversion in Nd^{3+}-sensitized core–shell nanoparticles. *J. Am. Chem. Soc.* **135**, 12608-12611 (2013).

9. Strohhöfer, C., & Polman, A. Absorption and emission spectroscopy in Er^{3+}–Yb^{3+} doped aluminum oxide waveguides. *Opt. Mat.* **21**, 705-712 (2003).
